# Testosterone eliminates strategic prosocial behavior through impacting choice consistency in healthy males

**DOI:** 10.1038/s41386-023-01570-y

**Published:** 2023-04-03

**Authors:** Hana H. Kutlikova, Lei Zhang, Christoph Eisenegger, Jack van Honk, Claus Lamm

**Affiliations:** 1 Department of Cognition, Emotion, and Methods in Psychology, University of Vienna, Vienna, Austria; 2 Department of Experimental Psychology, Helmholtz Institute, Utrecht University, Utrecht, the Netherlands; 3 Centre for Human Brain Health, School of Psychology, University of Birmingham, Birmingham, UK; 4 Institute for Mental Health, School of Psychology, University of Birmingham, Birmingham, UK; 5 Department of Psychiatry and Mental Health, University of Cape Town, Cape Town, South Africa; 6 Vienna Cognitive Science Hub, University of Vienna, Vienna, Austria

**Keywords:** Human behaviour, Gonadal hormones, Reward

## Abstract

Humans are strategically more prosocial when their actions are being watched by others than when they act alone. Using a psychopharmacogenetic approach, we investigated the endocrinological and computational mechanisms of such audience-driven prosociality. One hundred and ninety-two male participants received either a single dose of testosterone (150 mg) or a placebo and performed a prosocial and self-benefitting reinforcement learning task. Crucially, the task was performed either in private or when being watched. Rival theories suggest that the hormone might either diminish or strengthen audience-dependent prosociality. We show that exogenous testosterone fully eliminated strategic, i.e., feigned, prosociality and thus decreased submission to audience expectations. We next performed reinforcement-learning drift-diffusion computational modeling to elucidate which latent aspects of decision-making testosterone acted on. The modeling revealed that testosterone compared to placebo did not deteriorate reinforcement learning per se. Rather, when being watched, the hormone altered the degree to which the learned information on choice value translated to action selection. Taken together, our study provides novel evidence of testosterone’s effects on implicit reward processing, through which it counteracts conformity and deceptive reputation strategies.

## Introduction

Humans behave more prosocially when their actions are watched by others [1]. This phenomenon has been demonstrated across a variety of social behaviors, such as blood donations [2], church offerings [3], or monetary donations to charitable organizations [4], and is often referred to as strategic prosociality [5], or the audience effect [6]. From an evolutionary perspective, making one’s generosity visible to others has an important signaling value, in that it advertises an individual’s qualities as a potential partner or a valuable group member [7]. In the present study, we propose and investigate whether the steroid hormone testosterone plays a crucial role in shaping such audience effects.

Research in the past decade has demonstrated that testosterone is implicated in a wide spectrum of socially dominant behaviors [8, 9]. Exogenous testosterone alleviates subordination to the dominance of others [10–12] and reduces the physiological response to being evaluated by others [13]. Given that enhanced submission to audience expectations has been associated with intense apprehension about social evaluation [14], one possible prediction is that testosterone administration will decrease audience effects.

Contrasting with this view, the hypothesis that testosterone drives status-seeking via reputation building rather than dominance [15, 16] would predict that based on the social context, testosterone might conditionally promote prosocial and especially socially desirable behavior to build up a reputation and increase status.

The present paper is the first that aimed to distinguish between these two alternatives of boosting one’s social status that testosterone may act on. One option is that, in line with the social dominance hypothesis [17], the hormone prioritizes dominant status-seeking and would hence diminish the submission to audience expectations. The other option is that testosterone primarily promotes reputable status-seeking [15, 16]. If true, the hormone could increase strategic prosocial behavior.

Through what neurobiological pathways could testosterone modulate such complex social behaviors? Previously, exogenous testosterone was found to increase dopamine levels in the rat ventral striatum [18], suggesting that the hormone exerts its effects through the modulation of dopaminergic activity in reward-related neural circuits. Besides this insight from animal research, testosterone and reward processing have also been linked in humans [19, 20]. It remains to be shown, though, which specific aspects of reward processing testosterone acts on. For one, during value learning, testosterone may influence the incorporation of so-called prediction errors (PE), which track the difference between predicted and actual outcome [21] and are encoded by the phasic activity of midbrain dopaminergic neurons projecting to the ventral striatum [22, 23]. Alternatively, testosterone may impact the conversion of the learned values into action selection, or the temporal dynamics of the evidence accumulation.

The present study thus not only aimed to investigate if testosterone influences strategic prosociality, but also whether this is achieved by impacting reward-related computations. We employed a novel modeling approach, by combining reinforcement learning with diffusion decision models (RLDDM). This provided a more comprehensive account of the latent processes involved in prosocial decision-making than previous separate RL and DDM approaches [21, 24]. Besides describing how subjective values of the choice options are learned through PEs (*learning rate parameters*) and converted to actions (*choice consistency parameter*), the new combination of reinforcement learning and diffusion decision modeling also enabled us to explore the temporal dynamics of these latent processes (*decision threshold and drift-scaling parameters*, see SM Table S4 for parameter description) [24].

**Fig. 1:**
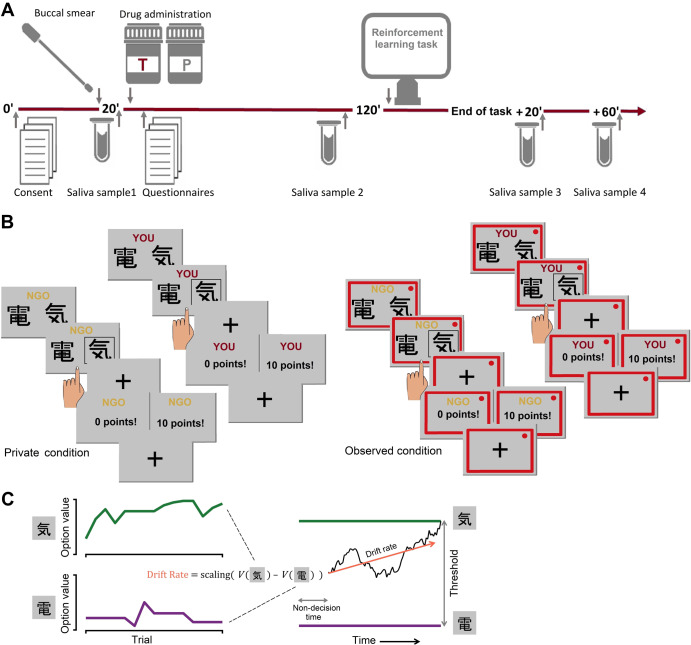
Experimental design and task. **A** Timeline of the experimental session. **B** Prosocial reinforcement learning task. Participants performed the task either in private or watched by an observer introduced as an NGO association representative. The observation was signaled by a red frame. Each participant completed three blocks of 16 trials for themself and three blocks of 16 trials to benefit an NGO of his choice. **C** Schematic of the reinforcement learning drift diffusion model (RLDDM). Left panel: trial-by-trial value updates in RL; right panel: evidence accumulation in DDM. Importantly, the drift rate in DDM is calculated from the value difference between choice options in RL.

Male participants underwent a double-blind, between-subject, placebo-controlled, testosterone administration and then performed a reinforcement learning task (Fig. 1). To compare self- and other-oriented decision-making, participants completed the task for themselves and for an NGO of their choice. While charitable donation tasks [16] and neuroeconomic games [8, 9] classically measure participants’ overall prosociality using deliberated decisions, such as deciding how much money to share with another person, the RL task allowed us to furthermore characterize the hidden individual steps in the process of learning about the consequences actions have for oneself and others (see [25, 26] for similar recent approaches). Critically, the task was performed either in private or when being watched (see “Materials and methods”).

Based on previous audience-effect research [2–6], we predicted that when the participants are watched, they will be relatively more prosocial (i.e., make more correct choices for the other vs self) than in private. Crucially, we expected that such an audience effect will be underpinned by relatively faster incorporation of the PEs (captured by the learning rate parameter α in RL); higher consistency in converting values to action probability (captured by the inverse temperature parameter tau in RL, also known as value sensitivity, exploration parameter, or 1/β); and more integrated evidence necessary for making a decision (captured by the threshold parameter in DDM). In other words, participants would learn more efficiently, learned values would inform their behavior more consistently, and their decisions would be more cautious.

Our main hypothesis was that the effects of being watched on other- vs. self-benefitting behavior will be modulated by testosterone administration. Given that testosterone reduces submission signals and stress responses to the social evaluation, allowing for dominant status-seeking [10–13], we hypothesized that testosterone would reduce the audience effect expected in the placebo group. As an alternative prediction, we reasoned that if testosterone does not primarily cause dominant status-seeking, but instead, in non-threatening environments, promotes more agreeable and reputable status-enhancing behaviors [15, 16], participants in the testosterone (vs placebo) group should show a larger audience effect. Irrespective of whether testosterone would increase or decrease prosocial behavior under the audience effect, we also predicted that testosterone’s effects will be associated with changes in the efficiency of PE-based value updating (α in RL), choice consistency (tau in RL), and evidence necessary for making a decision (threshold parameter in DDM).

Previous research has suggested that testosterone possibly modulates social behavior through both androgenic and dopaminergic pathways and that testosterone effects on status-seeking behavior are moderated by CAG repeat polymorphism of the androgen receptor [27], and DAT1 polymorphism of the dopamine transporter [28]. We, therefore, examined whether these polymorphisms interact with testosterone administration effects on strategic prosociality. Furthermore, as research has shown that testosterone effects on status-seeking and decision-making are influenced by endogenous cortisol levels [29, 30], we examined whether testosterone administration effects interact with salivary cortisol levels measured at baseline and with cortisol reactivity to being watched. Moreover, as it has been suggested that sensitivity of dopaminergic pathways is heightened among highly dominant individuals [31] and that these individuals show more pronounced effects of testosterone administration [27, 32], we tested whether testosterone effects on strategic prosociality vary as a function of self-reported trait dominance [33]. Lastly, we sought to shed light on the motivational aspects [15, 34] of testosterone’s actions. We, therefore, conducted an exploratory post-hoc analysis that tested whether testosterone effects on strategic prosociality interact with self-reported value system, based on a questionnaire that captures the motivational bases of human attitudes and behavior [35, 36].

## Materials and methods

### Participants

The study sample consisted of 192 healthy adult men aged between 18 and 40 years (*M* = 24.89, *SD* = 4.08). The sample size was determined based on previous testosterone administration studies [8, 13, 32] and our pilot study. In the applied linear mixed models, our sample size gave us 90% power to detect three-way and 86% power to detect 4-way interaction effects of size *f* ≥ 0.15. Using Monte Carlo simulation [37], we also calculated the probability of detecting a significant effect in the generalized linear mixed models (GzLMM) given our sample, experimental design, and the expected effect size based on previous research (*B* = 0.231) [16, 28]. The probability of a significant 3-way interaction was 93.68% (95% CI [89.08, 98.28]) and the probability of a significant 4-way interaction was 88.40% (95% CI [86.87, 89.31]. Participants were recruited via flyers placed around university campuses and online advertisements. The exclusion criteria comprised a history of neurological or psychiatric disorders, endocrine or other internal diseases, substance dependence, body mass index outside the healthy weight range (18.5–24.9), and the use of steroids. Only male participants were included as testosterone metabolism is subject to sex differences and the pharmacokinetics of topical administration of testosterone are unclear in women [38]. Two participants were excluded from the original sample *N* = 192 because they continually clicked on the same response key irrespective of changing stimuli and reward probabilities for more than 80% of the block trials, and thus were classified as non-compliant. This led to a final sample size *N* = 190. All participants gave written consent and received a financial reward for their participation consisting of a flat fee and a bonus based on their task performance. All procedures were approved by the Ethics committee of the Medical University of Vienna and conducted following the latest revision of the Declaration of Helsinki [39]. No side-effects or adverse events were reported during or after the experimental sessions.

### Procedure and experimental design

Testing took place in groups of three to five participants, who were seated individually in small cubicles within the same testing room. All experimental sessions started between 01:00 and 02:30 p.m. First, a buccal smear sample for CAG repeat and DAT1 polymorphisms analysis was taken (see SM: Supplementary information*on the analysis of genetic data*). 20 min after arrival, participants were asked to drool 2 mL of saliva into a polyethylene collection tube. All salivary samples were frozen on-site and stored at −30 °C until analysis. Afterward, participants were administered topical testosterone or placebo gel in a double-blind between-subjects design with random group allocation. Those allocated to the testosterone group received a single dose of testosterone gel containing 150 mg testosterone [Androgel®]; participants in the placebo group received an equivalent amount of placebo gel. The only difference between the testosterone and placebo gel was that the placebo gel did not contain testosterone. Participants rubbed the gel onto their upper arms and shoulders using disposable latex gloves. Gel administration was followed by a 2-h waiting period, during which participants remained in the laboratory premises, completed personality and demographic questionnaires, and were offered leisure-time reading materials. The testosterone dose and timing of the experiment were based on the previously established pharmacokinetic study of testosterone gel preparations in healthy young males [38]. One hour and 50 min after the gel application, participants provided a second saliva sample and subsequently began the experimental task (see Fig. 1). Two more saliva samples were taken during the course of the study: 20 and 60 min after the end of the experimental task. After data collection was complete, saliva samples were analyzed by liquid chromatography-tandem mass spectrometry.

Participants performed the experimental task under one of the two randomly assigned between-subject conditions: either in private or when being observed. In the private condition, participants were informed that their performance is completely anonymous and no one (including the experimenter) would know how much money they would earn for themselves and the charitable organization. In the observed condition, two female observers, introduced as NGO association representatives entered the room and watched participants perform the task. The observers were seated at a desk with a laptop and had an equal view of all the participants. In addition, when participants were observed, a red frame was shown on their computer’s screen and the displays of the participants’ computers were transmitted onto the observers‘ laptop screens.

Participants were thus randomly assigned into four experimental groups corresponding to the levels of two between-subject factors: (1) treatment (testosterone/placebo) and (2) visibility (observed/private). These groups did not differ in age, trait dominance, basal hormone levels, or distribution of AR CAG and DAT1 genotype (see SM: Table S1).

### Prosocial learning task

Participants performed a probabilistic reinforcement learning task [25], where they could earn rewards either for themselves (*self* condition) or for an NGO of their choice (*other* condition). On each trial, participants were presented with two abstract symbols, one associated with a high (75%) and the other with a low (25%) reward probability. These contingencies were not instructed but had to be learned through trial and error. Participants selected a symbol by a button press and then received feedback on whether they obtained points or not. This way participants learned which symbol to choose to maximize the rewards in the long run. The points were converted to monetary rewards at the end of the experiment. Participants completed 6 blocks, 3 blocks in self and 3 blocks in the other condition. Each block started with a new pair of symbols and consisted of 16 trials/choices. In the self-condition, the blocks started with “YOU” displayed and had the word “YOU” at the top of each screen. In the other condition, the blocks started with “NGO” displayed and had the word “NGO” at the top of each screen. The order of the blocks was pseudo-randomized so that the same recipient block did not occur twice in a row, and that half of the participants’ sample started the task with the *self* condition and the other half with the *other* condition. At the end of the experimental task, participants could choose the recipient of the money they earned in the *other* condition from a list of 6 different charities.

Immediately after completing the task, participants were asked to fill in a post-task questionnaire to estimate their subjective perception of being watched. The participants were asked the question: “Did you feel that you were being watched while performing the task?” The answers were classified into three categories: 1 (not at all), 2 (moderately), and 3 (strongly).

### Statistical analysis of correct choices

Statistical analysis was performed using R statistical language [40]. We analyzed the treatment (testosterone/placebo) × visibility (observed/private) × recipient (self/other) interaction effect on correct choice using generalized linear mixed models (GzLMM) with binomial distribution and logit link function. The correct choice was defined as choosing the symbol with a higher reward probability. The participant’s identity was modeled as a random intercept effect and the within-subject factor recipient (self/other) was entered as a random slope.

To examine whether the effects of testosterone on correct choice varied as a function of trait dominance, CAG repeat, DAT1 polymorphism, baseline cortisol, cortisol reactivity, and personal value orientations, we added these variables separately as predictors in interaction with the other factors specified in the above GzLMM (See SM for information on the used R packages).

### Reinforcement learning drift-diffusion modeling

To uncover the cognitive computational processes underlying our learning task, we performed modeling analysis under the joint reinforcement learning drift diffusion model (RLDDM) framework [24, 41]. In essence, RLDDM bridges RL, which typically models choices, and DDM, which commonly models response times (RT). This approach has been proven to provide more granularity than using RL or DDM alone [24]. We tested candidate models with a single learning rate (Rescorla-Wagner models) as well as models with dual learning rates. Together, we tested 6 candidate RLDDM models, and the winning model is described below (see SM: Supplementary information*on computational modeling* for full model description, estimation, and comparison procedures). The RL part of the winning RLDDM model was implemented with a dual learning rates reinforcement learning model, where both the learning rate for a positive prediction error and the learning rate for a negative prediction error were employed to update values (i.e., *V*(A) and *V*(B) for two-choice options) [42] (Eq. (1); see also SM: Supplementary information*on computational modeling)*.

The DDM part of the winning RLDDM model was implemented via a non-linear transformation of the accuracy-codded value differences computed from the RL counterpart, to construct the trial-by-trial drift rates [24] (Eq. (2); see also SM: Supplementary information*on computational modeling*). The winning model contained 14 parameters: 7 separate parameters for each between-subject condition (i.e., placebo/testosterone, private/observed), and differential parameters for the within-subject condition (i.e., other/self; see SM: Table S4 for the parameter list and description).

In all models, we simultaneously modeled participants’ choice and RT, separately for each between-subject condition (i.e., placebo/testosterone; observed/private). Model estimations of all candidate models were performed with hierarchical Bayesian analysis (HBA). HBA was particularly useful when the number of trials was limited (here 16 trials per block) because in HBA, group-level and individual-level parameters were mutually informing each other during model estimation. All models reached convergence (see SM: Supplementary information*on computational modeling*).

### Statistical analysis of model parameters

The drug treatment (P/T) × visibility (observed/private) × recipient (other/self) effect on the extracted free parameters was analyzed using GzLMMs analogous to the analysis of the correct choice. Due to the non-normal distribution of residuals, gamma distribution with a log link function was used for the parameter analyses. Finally, we tested whether the RLDDM parameter estimates, which were affected by the interaction of the drug treatment, visibility, and recipient could explain the differences observed in the behavioral prosociality measure. To do so, we conducted multiple linear regressions with the difference in the number of correct choices made for other and self (*prosociality index*) as a dependent variable and the differences in the RLDDM parameter estimates (αneg_other_- αneg_self_, *τ*_other_ − *τ*_self_, threshold_other_-threshold_self_, drift-scaling_other_-drift-scaling_self_) as separate predictors. Bonferroni correction for multiple comparisons was used.

## Results

### Manipulation check

In the testosterone, compared to the placebo group, we observed higher salivary testosterone levels 110 min after gel administration, and this difference remained stable until the end of the experiment (drug treatment x time: *F*(3, 554.82) = 48.00, *p* = 0.001, *R*^*2*^_conditional_ = 0.737), see Fig. S1 and SM: Supplementary analysis*of hormone data* for analysis details). Analysis of the subjective ratings of being watched showed that participants in the observed condition felt watched to a greater extent than in the private condition (*Χ*^2^ (2, *N* = 190) = 114.49, *p* < .001), and that testosterone administration did not influence the perception of being watched (*Χ*^2^ (2, *N* = 190) = 0.006, *p* = 0.997). The degree to which the participants felt watched, was positively associated *r* (185) = 0.177, *p* = 0.016) with cortisol reactivity to the visibility manipulation (Δ cortisol levels 20 min after the end of the task - cortisol levels immediately before the task).

### Testosterone eliminates the audience effect

**Fig. 2:**
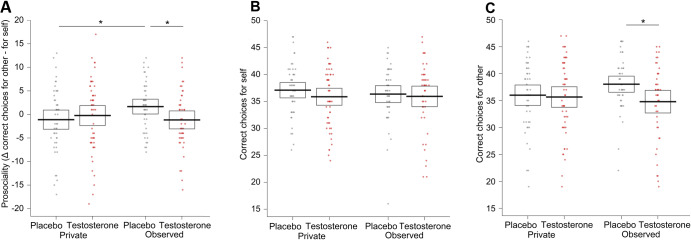
Differences in the number of correct choices. **A** Three-way interaction of the factors treatment × recipient × visibility. Participants in the placebo group behaved more prosocially (as captured by the prosociality index = correct choices for other – correct choices for self) when being observed than in privacy. Exogenous testosterone eliminated this audience effect. Note that the analyses were performed with raw data in the 2 × 2 × 2 factorial design; the plotted difference score (other minus self) is to improve readability and interpretability. Breaking down the three-way interaction by using treatment contrasts showed that there was no significant effect of the drug treatment and visibility factors, or their interaction, on the number of correct choices made for oneself (**B**). On the other hand, testosterone, compared to placebo, decreased the number of correct choices made for the NGO when being observed (**C**). Dots represent the data of individual participants, lines represent mean values per group, and boxes 95% confidence intervals.

The three-way interaction of the factors drug treatment (P/T), visibility (private/observed), and type of recipient (self/other) predicted the number of correct choices (i.e., options that have higher reward probability) the participants made (OR = 0.94, CI = [0.89, 1.00], *p* = 0.043; Fig. 2A). Follow-up analysis using treatment contrasts showed that participants in the placebo group showed more prosocial behavior, as indicated by relatively more correct prosocial choices when being watched compared to the private setting in which they were not watched (recipient × visibility interaction in the placebo group: OR = 1.43, CI = [1.01, 2.02], *p* = 0.042). Supporting our prediction based on the social dominance hypothesis, this audience effect was absent in the testosterone group (recipient x visibility interaction in the testosterone group: OR = 0.87, CI = [0.62, 1.22], *p* = 0.418). Specifically, when participants were observed, testosterone, compared to placebo, reduced the number of correct choices made for another (OR = 0.69, CI = [0.50, 0.94], *p* = 0.019, Fig. 2C). The number of correct choices made for self, however, was not influenced by the drug treatment (OR = 0.98, CI = [0.75, 1.28], *p* = 0.875), visibility (OR = 1.00, CI = [0.78, 1.30], *p* = 0.982, or their interaction (OR = 0.88, CI = [0.61, 1.28], *p* = 0.509, Fig. 2B).

### Behavior is best explained by a reinforcement learning drift diffusion model with dual learning rates

Next, we sought to uncover the computational mechanisms underlying the experiment-condition-specific behavioral differences on a trial-by-trial basis. The winning model (winning over five other candidate models; see “Materials and methods”, SM: *Model selection and validation*, and Table S3) entailed combined RL and DDM components, and thus simultaneously predicted individuals’ choices and RTs (see SM: Table S4 for a complete list of parameters and their description). The RL component section predicted participants’ learning behavior via the value updates through the computation of PEs with separate learning rates for positive and negative PEs (i.e., α^pos^ and α^neg^ Eq. (1)). In other words, the model that best accounted for the data assumed a differential speed of learning with and without positive feedback:

1 $$V_{c,t} = \left\{ \begin{array}{l}{{V_{c,t - 1} + \alpha ^{{{{{\rm{pos}}}}}}\left( {O_{i - 1} - V_{c,t - 1}} \right),\,{{{{{{{\mathrm{if}}}}}}}}\,{\it{O}}_{{\it{t - }}1} \, > \, 0}}\\ {{V_{c,t - 1} + \alpha ^{{{{{\rm{neg}}}}}}\left( {O_{i - 1} - V_{c,t - 1}} \right),\,{{{{{\rm{otherwise}}}}}}}}\end{array}\right.$$

where *O*_*t*-1_ denotes the outcome, and *V*_*c,t*-1_ the subjective value of choice *c* at trial *t−*1.

In addition, the DDM component predicted RTs by assuming an evidence accumulation process (as quantified by the drift rate; decisions were made when the evidence reached a certain threshold [41]). Importantly, the marriage between RL and DDM allowed a fine-grained investigation into how the drift rate (*v*_*t*_) was shaped by the value difference between two symbols at the trial-by-trial level (Eq. (2); *S*, a non-linear transformation function; *v*_scaling_, a weight parameter that maps accuracy-coded value difference into the drift rate [24]).

2 $$\upsilon _t = S\left[ {\upsilon _{{{{{{\rm{scaling}}}}}}}\left( {V_{{{{{{{{\mathrm{correct,}}}}}}}}{\it{t}}} - V_{{{{{{{{\mathrm{incorrect}}}}}}}},t}} \right)} \right]$$

We fitted all candidate models (see “Materials and methods” and SM: Supplementary information*on computational modeling*) under the hierarchical Bayesian estimation scheme [43] to incorporate both group-level commonality and individual differences, according to our task design (effects of drug treatment (P/T), visibility (private/observed), and type of recipient (self/other)).

### Testosterone’s impact on strategic prosocial behavior is associated with choice consistency

Next, we investigated which RLDDM parameters of our validated winning model are associated with the effects found in the behavioral analysis of the correct choice. As a first step, we tested the parameters for the 3-way interaction effect of drug treatment, visibility, and type of recipient.

**Fig. 3:**
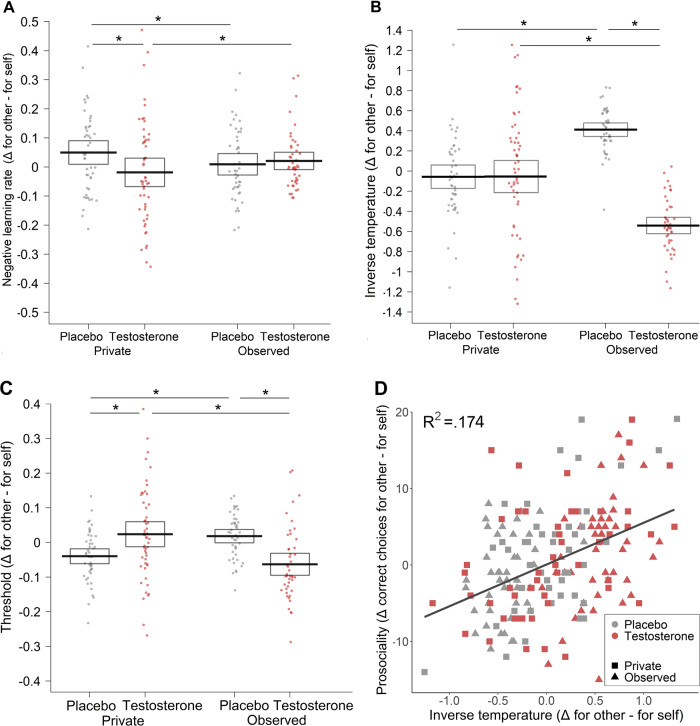
Differences in the parameters estimated by the reinforcement learning drift diffusion model (RLDDM). **A** In the placebo group, observation compared to privacy relatively decreased the prosocial learning rate for negative PE (i.e., the difference between α^negPE^ in the other condition and α^negPE^ in the self-condition). Testosterone administration reversed the observation effect. The results suggest that for better performance in the task, a lower learning rate from negative PE is more suitable. **B** In the placebo group, observation compared to privacy, relatively increased the consistency of the prosocial choices. Testosterone administration reversed this audience effect. **C** In the placebo group, observation compared to privacy, relatively increased the DDM threshold for prosocial choices. Testosterone administration reversed the audience effect. **D** Inverse temperature parameter tau that captures choice consistency significantly predicted prosociality. Dots represent the data of individual participants, lines represent mean values per group, and boxes 95% confidence intervals.

In the second step, we examined whether the parameters that showed a three-way interaction effect of our experimental manipulation predict behavioral prosociality (the difference between correct choices made for others and self). Out of the five parameters (learning rate for positive PE, learning rate for negative PE, choice consistency, threshold, drift-scaling parameter), only choice consistency showed the requisite three-way interaction of our experimental manipulations (*B* = 0.98, CI = [0.97, 0.98], *p* < 0.001), and at the same time significantly predicted behavioral prosociality (Bonferroni correction for multiple comparisons, *B* = 3.82, CI = [2.64, 5.01], *p* < 0.001; Fig. 3D). Specifically, participants in the placebo group displayed relatively higher consistency in choices made for the other (vs. self) when being observed than in privacy (recipient × visibility interaction in the placebo group: *B* = 1.09, CI = [1.05, 1.14], *p* < 0.001). On the contrary, in the testosterone group, observation, compared to privacy, decreased the consistency of choices made for the other (vs. self) (recipient × visibility interaction in testosterone group: *B* = 0.90, CI = [0.84, 0.98], *p* < 0.001; Fig. 3B). When participants were observed, testosterone, compared to placebo, diminished the relative consistency of prosocial choices (recipient × treatment interaction in observed condition: *OR* = 0.91, CI = [0.84, 0.99], *p* = 0.025; for analysis of all RLDDM parameters, see SM: *Analysis of the RLDDM parameters and their association with prosocial behavior*). Altogether, these results suggest that testosterone eliminates audience-dependent prosocial behavior by affecting choice consistency.

### Interaction of testosterone effects with trait dominance

In further support of the social dominance hypothesis, trait dominance interacted with testosterone’s effects on correct choice (recipient × drug treatment × visibility × trait dominance: OR = 1.04, CI = [1.01, 1.09], *p* = 0.026). In a follow-up analysis aimed at decoding this interaction, the continuous measure of dominance was replaced by a categorical variable with levels of high and low dominance, based on the median split of dominance scores (Med = 3.949). Decomposition of the four-way interaction revealed that testosterone reduced the number of correct choices made for others during observation specifically among men with high trait dominance (OR = 0.60, CI = [0.42, 0.87], *p* = 0.008) and this effect was weaker and non-significant among those with low dominance (OR = 0.77, CI = [0.55, 1.04], *p* = 0.132). Trait dominance did not significantly interact with the RLDDM parameters (all *p*s > 0.331 see SM: *Interaction of trait dominance with testosterone effects on RLDDM parameters*).

### Interaction of testosterone effects with genetic polymorphisms and cortisol levels

CAG-repeat and DAT1 polymorphisms did not interact with the effects of testosterone on correct choice or RLDDM parameters (all *p*s > 0.091, see SM: Supplementary information on the analysis of genetic data). Furthermore, neither baseline cortisol levels, nor cortisol reactivity to visibility manipulation (Δ cortisol levels 20 min after the end of task - cortisol levels immediately before the task) interacted with the effects of testosterone on correct choice or RLDDM parameters (all *p*s > 0.052, see SM: Supplementary analysis of hormone data, Fig. S2*, and* Table S2*for details on cortisol levels*).

### Interaction of testosterone effects with personal value orientations

We next explored whether four principal value orientations (self-enhancement, self-transcendence, openness, conservation) measured using a self-report questionnaire [35, 36] interacted with testosterone’s influence on the number of correct choices and choice consistency.

This revealed an interaction of *self-enhancement value orientation* with testosterone’s effect on the number of correct choices (recipient × visibility × administration × self-enhancement: OR = 0.65, CI = [0.48, 0.88], *p* = 0.006) and interaction of *conservation value orientation* and testosterone’s effects on choice consistency (recipient × visibility × administration × conservation: *B* = 0.60, CI = [0.17, 1.02], *p* = 0.006). The treatment contrast analysis of the interaction effect with self-enhancement showed that when testosterone participants were watched, a higher score in self-enhancement value was negatively associated with correct prosocial choices (OR = 1.29, CI = [0.21, 2.36], *p* = 0.008). In the placebo group, we did not observe such an association (OR = 1.11, CI = [0.89, 1.38], *p* = 0.349) and this difference between testosterone and placebo group was significant (OR = 0.72, CI = [0.55, 0.94], *p* = 0.018).

The treatment contrast analysis of the interaction effect with conservation value showed that when placebo participants were watched, the prosocial choice consistency was positively associated with conservation value (*B* = 1.29, CI = [0.22, 2.35], *p* = 0.019). However, after testosterone administration, the link between conservation value and prosocial choice consistency was abolished (*B* = −0.02, CI = [−0.94, 0.90, *p* = 0.969), but this difference between testosterone and placebo group did not reach significance (OR = 1.30, CI = [−0.10, 2.71], *p* = 0.069). No other interaction between value orientations and testosterone effect on choice behavior or RLDDM parameters was detected (all *p*s > 0.287 see SM: Supplementary information*on the questionnaire data)*.

### Learning parameters in relation to optimal learning rates

**Fig. 4:**
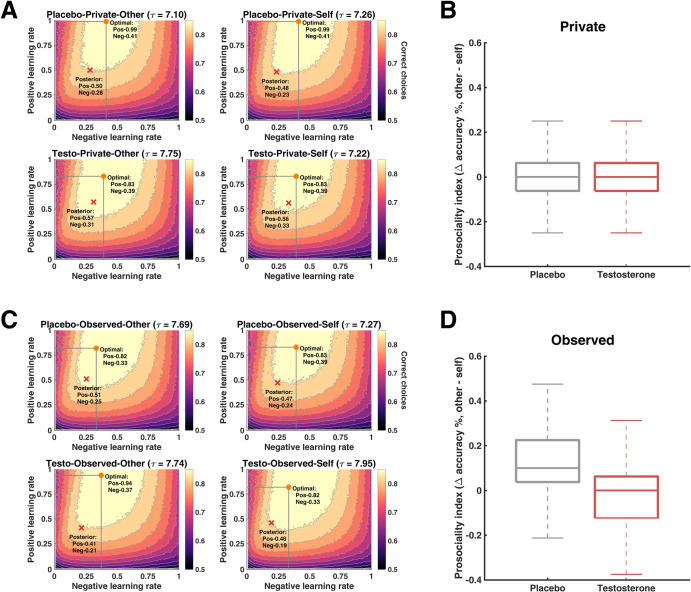
Optimal learning rates and posterior predictive checks. Posterior learning rates in relation to the optimal learning rates in the private (**A**) and observed (**C**) conditions. Orange dots represent the optimal combination between learning rates for positive and negative PE identified via simulation; red crosses indicate the posterior means of learning rates. The posterior learning rates were employed to perform posterior predictive checks for the main behavioral findings for the private (**B**) and observed (**D**) conditions. Simulated data from posteriors were analyzed in a similar fashion as the real data and the model prediction largely matched our main behavioral effect (cf. Fig. 2A).

To gain a deeper understanding of how the learning parameters were related to the task performance in our experimental design, we performed a simulation study to identify optimal learning rates [44] (see SM: *Simulations of optimal learning rates*). In all conditions, both the posterior positive and negative learning rates were smaller with respect to the optimal ones (see Fig. 4A, C). Crucially, to validate whether the choice accuracy corresponding to the posterior parameters in our winning model could capture key patterns in our behavior findings (i.e., posterior predictive check), we let our winning model generate synthetic data and analyzed the generated prosocial behavior (i.e., choice accuracy for other minus choice accuracy for self) in the same way as we analyzed the observed data. We found that results from the generated data (Fig. 4B, D) greatly resembled the behavioral patterns reported in Fig. 2A.

## Discussion

Using pharmacological manipulation and a novel computational model integrating reinforcement learning with the drift diffusion modeling framework (RLDDM), we tested and characterized testosterone’s role in audience-dependent prosocial behavior. The results show that testosterone diminishes the typical audience effect present in the placebo condition. Computational modeling pinpoints this effect to a reduction in the extent to which the performance of prosocial (vs. selfish) choices is consistent with learned reward values. Moreover, the effects are more pronounced in participants with higher trait dominance. Taken together, these findings are in line with the social dominance hypothesis and are thus consistent with the notion that testosterone decreases submission to audience expectations, rather than promoting the strategic display of socially pleasing behavior [45, 46].

A growing body of evidence suggests that testosterone exerts its behavioral effects through the modulation of reward-related processes [19, 20]. However, to our knowledge, no study investigated the computational mechanisms underlying such effects. Using joint RLDDMs, we found that in the placebo group, observation (vs privacy) increased the relative consistency of prosocial choices. Testosterone administration eliminated this audience effect, making the performance of prosocial (vs. self-benefitting) choices less consistent with value computations. Low choice consistency means that individuals select options with non-maximal expected values, which is often referred to as exploratory behavior [47]. In environments with static reward probabilities, participants can maximize their reward by initially exploring which option tends to be more fruitful. Once learners discover the better option, exploration yields no benefit. One possible explanation of the present effect could therefore be that testosterone impaired individuals’ ability to adapt and control the amount of exploration. However, our data do not indicate that testosterone affects exploration in general, as we did not find any testosterone influence on choice consistency in the private setting. Instead, the effects of testosterone appeared only in a situation where social status was at stake. Interestingly, research in monkeys has shown that socially challenging environments (vs socially isolated environments) increased the availability of D_2_ receptors [48], which play a role in choice consistency [49]. This effect was present in particular in dominant monkeys and absent in the subordinate ones. Such findings are in line with our results showing enhanced testosterone effects in individuals with high trait dominance. We, therefore, suggest that future studies should examine the potential mediating role of D2 receptors in testosterone’s effect on reward learning in social situations.

Alternatively, it could be speculated that the elimination of the audience effect by testosterone stems from the hormone’s ability to reduce fear in social situations. Indeed, earlier research shows that exogenous testosterone diminishes the physiological stress response to the presence of an observer [13] and has anxiolytic-like properties in humans and across species [10, 50]. However, we did not observe any interaction of the testosterone’s effect with cortisol levels measured at baseline or with cortisol reactivity, thus our data do not provide support for such an interpretation. To examine this topic further, studies on testosterone and audience effect should include more explicit measures of subjectively perceived stress and anxiety.

The present data also shows that testosterone administration substantially alters the relation between the audience effect and self-reported value orientations. *Self-enhancement* value, as measured here [35], is characterized by power, achievement, and the pursuit of one’s own interests, success, and dominance over others. We found that a higher emphasis on self-enhancement was related to a lower number of correct prosocial choices while being watched in the testosterone, but not in the placebo group. This finding extends the evidence that the hormone testosterone interacts with dominant personality traits. In contrast to self-enhancement, conservation value reflects a personal emphasis on conformity, security, tradition, and self-restriction [35]. We showed that in the placebo group, the degree to which one identifies with conservation value is positively associated with the consistency of prosocial choices made while being watched. Testosterone administration abolished this link between conservation value and prosocial choice consistency. In consideration of the conformity aspect of the conservation value, it is of interest that in Western societies, nonconforming people are perceived as having higher status and competence than those who conform to the social expectations [51]. Thus, these findings are suggestive of the interpretation that by inducing a non-confirming attitude, testosterone reaches its principle goal - to be observed as having high status and competence [17, 46]. We note though that the analyses of value orientations are exploratory and were performed post-hoc, and thus need independent confirmation.

Variability in dominance, conservation, and cultural differences in social status attaintment, can also account for the results of another recent study, which was conducted among Chinese students and showed that testosterone enhanced audience effects [16]. Indeed, contrary to Western society, in Eastern cultures, high social status is associated with increased self-restriction and other-orientation [52]. Consistently, individuals from Western and Eastern cultures differ in their self-construal, i.e., in the way they define the self in relation to others, with, for example, European individuals construing themselves as being more independent, or less interdependent, than Asian individuals [53]. Interestingly, research has shown that acute testosterone changes in men are positively associated with aggressive behavior for those with more independent self-construals, whereas basal testosterone is negatively associated with aggression when individuals have more interdependent self-construals [54]. Moreover, the cultural differences in independent vs. interdependent social orientations have been linked to polymorphisms in the dopamine D4 receptor gene [53], implying a putative biological mechanism that could explain cultural differences in testosterone effects. Finally, contrasting results may also stem from differences in the applied methods. While Wu et al. [16] used a modified dictator game, where participants were explicitly asked whether they want to donate a certain monetary amount to charity, in the present task, the donation to charity was determined indirectly by the participant’s performance in a reinforcement learning task. Our paradigm thus presents a more implicit measure of prosociality. For these reasons, we suggest future studies of how testosterone affects status-seeking behavior should focus on exploring these cultural differences, specifically by including measures of value orientations, self-construal, implicit behavioral tasks, as well as assessments of dopamine receptor polymorphisms.

Our results are, furthermore, in line with studies showing that testosterone decreases deception [55–57]. Further research is, however, needed to determine whether testosterone reduces lying per se, or only in situations where dishonest behavior may be considered “cheap”, dishonorable, and lower the subject’s feelings of pride and self-image [55].

There are also some limitations inherent to the methodology of our study. Due to the sex differences in testosterone metabolism and unknown pharmacokinetics following the topical administration of testosterone in women [38], the study included only male participants. Hence, the generalization of these findings to females requires further investigation. Furthermore, the observers in our study were exclusively female. Future research will also be required to more systematically test whether testosterone’s influence on the prosocial audience effect is sensitive to the gender, number, and salience of the present observers. Nevertheless, our survey data suggest that the salience of the observers used in our study was representative of a wide range of social contacts and that the subjective feeling of being watched scaled with an established stress biomarker.

In conclusion, we conducted a multifaceted examination of the computational, endocrinological, and genetic mechanisms underlying audience effect and showed that testosterone reduced strategic prosocial learning through impairment of choice consistency. These findings provide evidence that in the Western student sample, testosterone abolishes audience effects, and therefore does not foster the seeking of social leadership by reputational politics. The present study is the first to specify testosterone’s role in reward processing by revealing that testosterone impacts status-seeking through modulation of how the learned reward values are expressed in behavior.

## Supplementary information

Supplemental Material
