# Correction: Testosterone eliminates strategic prosocial behavior through impacting choice consistency in healthy males

**DOI:** 10.1038/s41386-023-01630-3

**Published:** 2023-06-29

**Authors:** Hana H. Kutlikova, Lei Zhang, Christoph Eisenegger, Jack van Honk, Claus Lamm

**Affiliations:** 1 Department of Cognition, Emotion, and Methods in Psychology, University of Vienna, Vienna, Austria; 2 Department of Experimental Psychology, Helmholtz Institute, Utrecht University, Utrecht, the Netherlands; 3 Centre for Human Brain Health, School of Psychology, University of Birmingham, Birmingham, UK; 4 Institute for Mental Health, School of Psychology, University of Birmingham, Birmingham, UK; 5 Department of Psychiatry and Mental Health, University of Cape Town, Cape Town, South Africa; 6 Vienna Cognitive Science Hub, University of Vienna, Vienna, Austria

**Keywords:** Human behaviour, Gonadal hormones, Reward

Correction to: *Neuropsychopharmacology* 10.1038/s41386-023-01570-y, published online 03 April 2023

The article “Testosterone eliminates strategic prosocial behavior through impacting choice consistency in healthy males”, written by Hana H. Kutlikova, Lei Zhang, Christoph Eisenegger, Jack van Honk and Claus Lamm was originally published electronically on the publisher’s internet portal on 3 April without open access. With the author(s)’ decision to opt for Open Choice the copyright of the article changed on 15 May 2023 to © The Authors 2023 and the article is forthwith distributed under a Creative Commons Attribution 4.0 International License, which permits use, sharing, adaptation, distribution and reproduction in any medium or format, as long as you give appropriate credit to the original author(s) and the source, provide a link to the Creative Commons licence, and indicate if changes were made. The images or other third party material in this article are included in the article’s Creative Commons licence, unless indicated otherwise in a credit line to the material. If material is not included in the article’s Creative Commons licence and your intended use is not permitted by statutory regulation or exceeds the permitted use, you will need to obtain permission directly from the copyright holder. To view a copy of this licence, visit http://creativecommons.org/ licenses/by/4.0/.

The original article has been corrected.

