# Supplementary material for: Testosterone eliminates strategic prosocial behavior through impacting choice consistency in healthy males
Source: Neuropsychopharmacology. 2023 Apr 3;48(10):1541–50. doi: 10.1038/s41386-023-01570-y (PMC10425362; doi:10.1038/s41386-023-01570-y)
Supplement: Supplementary file 1 — Supplemental Material [file 41386_2023_1570_MOESM1_ESM.pdf]

Supplementary Materials for:

Testosterone eliminates strategic prosocial behavior through impacting choice consistency in healthy males

Hana H. Kutlikova, Lei Zhang, Christoph Eisenegger, Jack van Honk, Claus Lamm

Corresponding author: Hana H. Kutlikova
Email: hana.kutlikova@univie.ac.at

This PDF file includes:

Supplementary text
- Supplementary information on the use of statistical software and functions
- Supplementary analysis of hormone data
- Supplementary information on computational modeling
- Supplementary information on the analysis of the genetic data
- Supplementary information on the questionnaire data

Figures S1, S2
Tables S1 to S4
SM References

Other supplementary materials for this manuscript include the following:
Data and codes can be accessed at: https://osf.io/qr4ve/

Supplementary information on the use of statistical software and functions

Statistical analysis was performed using the R statistical language [1] and the packages: lme4 [2] for construction of general linear mixed models (GLMM) and generalized linear mixed model (GzLMM); car package [3] for construction of general linear models and computation of *p*-values based on Type III Wald chi-square tests; sjPlot package [4] for post-hoc tests of significant three-way interactions including odds ratios (ORs) and 95% confidence intervals (95%CIs); yarrr [5] and ggplot2 [6] packages for construction of plots. Computational modeling was performed using Markov chain Monte Carlo with the statistical computing language Stan [7] while following the hBayesDM package [8]. Model comparison and evaluation were performed using the LOO package [9].

Supplementary analysis of hormone data

The effect of drug treatment on hormone levels.

After data collection was complete, saliva samples were shipped on dry ice to Dresden LabService GmbH led by Clemens Kirschbaum, Germany. Liquid chromatography-tandem mass spectrometry was used to determine the hormonal levels. To examine the change of the hormonal levels throughout the experimental session, testosterone, cortisol, and estradiol levels were analyzed using GLMMs with the fixed factors drug treatment (testosterone/placebo), visibility (observed/private), time (baseline/1 h 50 min after drug treatment/20 min after the end of the task/60 min after the end of the task), and participant’s identity as a random intercept. Due to the non-normal distribution of residuals, hormonal data were log-transformed. Baseline hormonal levels did not significantly differ across experimental groups (all *p*s > .336, see Table S1). As expected, 1h 50 min after gel administration, we observed higher testosterone levels in the testosterone group ($M_{Sample2}$ = 5014.10 pg/mL, 95%CI [3866.10, 6502.88]) compared to the placebo group ($M_{Sample2}$ = 134.30, 95%CI [103.54, 175.92]; drug treatment x time: $F(3,554.82) = 48.00$, $p < .001$, $R^2_{conditional}$ =.737), a difference that remained stable until the end of the experiment (see Figure S1).

There were no effects of drug treatment on cortisol (drug treatment x time: $F(3,520.6) = 1.246$, $p = .292$) or estradiol levels (drug treatment x time: $F(3,550.79) = 1.497$, $p = .214$).

The observation condition did not significantly influence any hormonal levels (testosterone: visibility x time: $F(3,554.82) = 2.447$, $p = .063$; cortisol: visibility x time: $F(3,520.6) = 0.900$, $p = 441$; estradiol: visibility x time: $F(3,550.79) = 0.796$, $p = .496$).

Contamination of salivary samples.

Out of a total of 192 baseline samples, we noted that 34 contained above-normal testosterone levels, atypical for normal young men (> 2000 pg/mL). All other baseline values were hormonally typical. The samples with abnormally high testosterone values appeared only in the participants, who later

received testosterone treatment, not in the placebo group. Previous research [10, 11] described similar abnormally high testosterone levels and attributed it to the testosterone contamination of the common surfaces (e.g., doorknobs, keyboards), excluding the option of physiological contamination. Based on their recommendation, we implemented a cleaning protocol that included the wearing of disposable sterile gloves, and cleaning of keyboards, computer mice, tables, and doorknobs with an alcohol-based solution after each session. Although these precautions successfully prevented between-session contamination, we suspect that they still did not reliably impede within-session contamination of the saliva containers. For future studies, we, therefore, recommend even stricter sanitizing protocols and more careful handling of the saliva collection tubes and boxes, before, during, and also after sample collection.

In our sample, the abnormally high values were present only in the sessions where testosterone was administered, and this notwithstanding, the testosterone group showed a reliable testosterone increase after the drug administration in comparison to the placebo group. We, therefore, decided to retain the participants with contaminated baseline samples for the behavioral analyses, except for the analysis that includes baseline testosterone levels.

The effect of salivary testosterone levels on the correct choice.

To examine whether salivary testosterone levels measured in the saliva samples taken before the start of the experimental task (i.e., 2 hours after the drug administration) predicted participants' behavior, we included the mean-centered log-transformed testosterone levels as a predictor in interaction with the factors recipient and visibility to the GzLMM of the correct choice. The analysis did not reveal a main effect of testosterone levels (*OR* = 0.99, *CI* = [0.96, 1.03], *p* = .624) or a significant interaction effect on correct choice (recipient x drug treatment x testosterone levels : *OR* = 1.02, *CI* = [0.99, 1.05], *p* = .162). The absence of a significant association between salivary testosterone concentrations and behavior is in line with studies that point out that although salivary testosterone measurements are correlated with the hormone concentration in serum, they do not precisely track the availability of free serum testosterone after transdermal application [11, 12]. Thus, although the between-groups comparison of saliva testosterone provides a manipulation check of topical drug administration, for the analysis of the relationship between behavior and post-administration hormonal levels on the individual level, salivary testosterone measures may presently lack sensitivity**.**

Interaction of salivary cortisol levels with testosterone effects on the correct choice and RLDDM parameters.

To examine whether cortisol levels interacted with testosterone's effect on correct choice and reinforcement learning drift diffusion model (RLDDM) parameters, we separately added baseline cortisol levels and cortisol reactivity as predictors to our main analysis. The cortisol reactivity was defined as a difference between cortisol levels detected in the saliva sample taken 20 minutes after the

end of visibility manipulation and cortisol levels from the sample taken immediately before the start of the paradigm. The values were mean-centered and entered as a predictor in interaction with the other factors (recipient, drug treatment, visibility) to the GzLMM of correct choice and the GzLMMs of the RLDDM parameters with log link function (see *Materials and Methods* in the main manuscript text). The analysis revealed no significant interaction of baseline cortisol levels with testosterone effect on correct choice (recipient x drug treatment x visibility baseline cortisol: $OR = 1.02$, $CI = [0.97, 1.07]$, $p = .385$), α^{posPE} (recipient x drug treatment x visibility baseline cortisol: $B = 1.01$, $CI = [0.98, 1.04]$, $p = .549$), α^{negPE} (recipient x drug treatment x visibility baseline cortisol: $B = 0.97$, $CI = [0.94, 1.00]$, $p = .052$), choice consistency (recipient x drug treatment x visibility baseline cortisol: $B = 1.00$, $CI = [0.99, 1.01]$, $p = .583$) or decision threshold (recipient x drug treatment x visibility baseline cortisol: $OR = 1.00$, $CI = [1.00, 1.0]$, $p = .902$).

Similarly, the analysis revealed no significant interaction of cortisol reactivity with testosterone effect on correct choice (recipient x drug treatment x visibility x post-task cortisol: $OR = 1.04$, $CI = [0.99, 1.09]$, $p = .133$), α^{posPE} (recipient x drug treatment x visibility x post-task cortisol: $B = 1.01$, $CI = [0.99, 1.03]$, $p = .454$), α^{negPE} (recipient x drug treatment x visibility x post-task cortisol: $B = 1.01$, $CI = [0.99, 1.01]$, $p = .078$), choice consistency (recipient x drug treatment x visibility x post-task cortisol: $B = 1.00$, $CI = [1.00, 1.01]$, $p = .249$) or decision threshold (recipient x drug treatment x visibility x post-task cortisol: $B = 1.00$, $CI = [1.00, 1.00]$, $p = .996$).

Supplementary information on computational modeling

Rescorla-Wagner (RW) model.

We started with the simple Rescorla-Wagner [13] model as our baseline model. On each trial, the value ($V_{c,t}$) of the chosen option was updated with the reward prediction error (RPE):

$$V_{c,t} = V_{c,t-1} + \alpha \left(O_{t-1} - V_{c,t-1}\right), \quad (1)$$

where O_{t-1} was the received outcome, and α ($0 < \alpha < 1$) denoted the learning rate.

Dual learning rates (DLR) model.

Previous studies have reported differences in learning following positive and negative PEs [14, 15] and these differences have been linked to the distinctive roles of striatal D1 and D2 dopamine receptors in segregated cortico-striatal pathways [16]. Furthermore, analyses of genetic polymorphisms of DARPP-32 that predict choice behavior associated with a positive outcome, and the DRD2 gene predicting avoidance of choices associated with negative outcomes, support the notion that independent dopaminergic mechanisms contribute to learning from positive and negative feedback [17]. RL models with dual learning rates have been successful in capturing this asymmetric

learning effect [18,19], including studies in social neuroscience examining prosocial behavior [20]. Hence, we tested a dual learning rates model on top of the RW model:

$$V_{c,t} = \begin{cases} V_{c,t-1} + \alpha^{\text{pos}} \left(O_{t-1} - V_{c,t-1} \right), \text{if } O_{t-1} > 0 \\ V_{c,t-1} + \alpha^{\text{neg}} \left(O_{t-1} - V_{c,t-1} \right), \text{otherwise} \end{cases}, \quad (2)$$

where α^{posPE} and α^{negPE} were the learning rates for positive and negative RPEs, respectively.

In both RW and DLR models, action values were converted to action probabilities using the softmax function. Let A and B be the choice symbols per trial, the probability of choosing A was computed via the difference between *V*(A) and *V*(B):

$$p(\text{A}) = \frac{1}{1 + e^{\beta(-(V_{\text{A}} - V_{\text{B}}))}}, p(\text{B}) = 1 - p(\text{A}), \quad (3)$$

where β (β > 0) was the inverse temperature that represented choice consistency. Higher β indicated that individuals' choices were more consistent with their value computation, where lower β indicated that individuals behaved more randomly. The action probability was then used to model participants' choice data with a categorical distribution:

$$\text{choice}_t \sim categorical([p_t(\text{A}), p_t(\text{B})]) \quad (4)$$

It is worth noting that our winning model with differential learning rates for positive and negative PEs is in line with previous studies on learning and decision-making that report asymmetric learning effect [14, 15, 18, 19]. The theoretical interpretation of these differences, however, is mixed in the literature. While some studies reported enhanced learning after positive PE compared to negative and related this feature to optimism bias [21], others report a higher learning rate for negative than for positive PEs, interpreted as possibly reflecting risk aversion [14]. Our findings in this regard may contribute to this debate such that learning update is much quicker after receiving positive feedback, especially when the feedback is associated with appetitive stimuli (e.g., monetary reward). This is likely to be generalized to situations with relatively positive feedback, rather than the actual positive feedback *per se*. For instance, in aversive learning, receiving no feedback (e.g., a neutral outcome) is, on a relative scale, more positive than actual negative feedback (e.g., an electric shock). Learning rates for such no-feedback events have been shown to be higher than those for stimuli with negative feedback, including learning under social contexts [20].

Drift diffusion model (DDM).

The drift diffusion model (a.k.a., diffusion decision model [22]) was a widely used computational framework to model individuals' response times (RTs). In its canonical expression, DDM contained four parameters, namely, the drift rate (v; v > 0), the initial bias (z; z > 0), non-decision time (T; 0 < T min (RT)), as well as the decision threshold (a). For simplicity in learning tasks with abstract symbols,

the initial bias z was fixed at 0.5. Trial-by-trial RTs were distributed according to the Wiener first passage time (WFPT [23]):

$$RT_t \sim wfpt(a, T, z, v) \quad (5)$$

Reinforcement learning drift diffusion model (RLDDM).

In value-based decision-making, individuals' RTs may vary as the function of trial-by-trial valuation, such that the larger the value difference between choice alternatives, the faster the RT. Therefore, a joint reinforcement learning drift diffusion model (RLDDM) framework has been proposed [15,24], bridging RL and DDM. This approach provides more granularity than using RL or DDM alone [24]. In essence, the drift rate in DDM was characterized by the accuracy-coded value differences computed from the RL counterpart. This way, the drift rate was no more a constant parameter throughout the entire experiment, instead, it varied across trials (i.e., v_t, instead of v) according to the values computed from RL updates (in the present study, RW or RP). In the simplest RLDDM, trial-by-trial drift rates were constructed via a linear function of value difference:

$$v_t = v_{\text{scaling}}\left(V_{\text{correct},t} - V_{\text{incorrect},t}\right), \quad (6)$$

where v_{scaling} ($v_{\text{scaling}} > 0$) was the scaling parameter that quantified the impact of value difference. Note that we employed stimulus coding in our RLDDM, so that in Equation (6), the drift rate was always a function of the value difference between the correct (i.e., more rewarding, 75% reward probability) and the incorrect options (i.e., less rewarding, 25% reward probability), rather than between the chosen and unchosen options.

Reinforcement learning drift diffusion model with non-linear transformation (RLDDM-nonlin).

There is evidence that a non-linear mapping between value difference and the drift rate could better capture individuals' RTs as opposed to a linear transformation [24]. This is likely because non-linear functions may provide more sensitivity, akin to the softmax function in choice models. We thus implemented an RLDDM-nonlin following:

$$v_t = S\left[v_{\text{scaling}}\left(V_{\text{correct},t} - V_{\text{incorrect},t}\right)\right], \quad (7)$$

with

$$S(x) = 2 \cdot \frac{v_{\max}}{1 + e^{-x}} - v_{\max}, \quad (8)$$

where $S(x)$ was a non-linear sigmoid function centered at 0, that convert x to lie between $-v_{\max}$ and $v_{\max}$ ($v_{\max} > 0$). It is worth noting that $v_{\max}$ only affected the maximum value of the drift rate, whereas v_{scaling}, as in Equation 6, established the trial-by-trial mapping between the value difference and the drift rate.

In both RLDDM and RLDDM-nonlin, all other DDM parameters (i.e., *a*, *T*, *z*) were identical to the canonical DDM model, and RTs were distributed with *wfpt* using trial-by-trial drift rate (v_t):

$$RT_t \sim wfpt(a, T, z, v_t) \quad (9)$$

Note that, in all candidate models (Table S3), we introduced differential parameters for the within-subject condition of our experiment, namely, all parameters were separately modeled for the "self" and the "other" conditions.

Model estimation.

The model estimation and model selection procedures were largely similar to [25]. Hence, below we echoed these procedures from [25] to enhance reproducibility, with modifications that were specific to the current study.

In all models, we simultaneously modeled participants' choice and RT, separately for each between-subject condition (i.e., placebo vs. testosterone; observed vs. private). Model estimations of all candidate models were performed with hierarchical Bayesian analysis (HBA) [26] using the statistical computing language Stan [7] in R. Stan utilizes a Hamiltonian Monte Carlo (HMC; an efficient Markov Chain Monte Carlo, MCMC) sampling scheme to perform full Bayesian inference and obtain the actual posterior distribution. We performed HBA rather than maximum likelihood estimation (MLE) because HBA provides much more stable and accurate estimates than MLE [24]. Following the approach in the "hBayesDM" package [8] for using Stan in the field of reinforcement learning, we assumed, for instance, that a generic individual-level parameter φ was drawn from a group-level normal distribution, namely, φ ~ Normal (μ_φ, σ_φ), with μ_φ and σ_φ. being the group-level mean and standard deviation, respectively. Both these group-level parameters were specified with weakly-informative priors [26]: μ_φ ~ Normal (0, 1) and σ_φ.~ half-Cauchy (0, 1). This was to ensure that the MCMC sampler traveled over a sufficiently wide range to sample the entire parameter space.

Appropriate parameter transformations were applied to double-bounded parameters (e.g., learning rate, [0, 1]) with the inverse probit function (i.e., the cumulative distribution function of the standard normal distribution), and single-bounded parameters (e.g., drift rate, (0, +∞)) with the soft-plus function (i.e., ln(1 + e^x)), respectively.

In HBA, all group-level parameters and individual-level parameters were simultaneously estimated through the Bayes' rule by incorporating behavioral data. We fit each candidate model with four independent MCMC chains using 1,000 iterations after 1,000 iterations for the initial algorithm warmup per chain, which resulted in 4,000 valid posterior samples. The convergence of MCMC chains was assessed both visually (from the trace plot) and through the Gelman-Rubin $\hat{R}$ Statistics [27]. $\hat{R}$ values of all parameters were smaller than 1.05 in the current study), which indicated adequate convergence.

Model selection and validation.

For model comparison and model selection, we computed the Leave-One-Out information criterion (LOOIC) score per candidate model [28]. The LOOIC score provides the point-wise estimate (using the entire posterior distribution) of out-of-sample predictive accuracy in a fully Bayesian way, which is more reliable compared to information criteria using point-estimate (e.g., Akaike information criterion, AIC; deviance information criterion, DIC). By convention, a lower LOOIC score indicates better out-of-sample prediction accuracy of the candidate model. We selected the model with the lowest LOOIC as the winning model. We additionally performed Bayesian model averaging (BMA) with Bayesian bootstrap [29]to compute the probability of each candidate model being the best model. Conventionally, the BMA probability of 0.8 (or higher) is a decisive indication.

Moreover, given that model comparison provided merely relative performance among candidate models [28], we then tested how well our winning model's posterior prediction was able to replicate the key features of the observed data (a.k.a., posterior predictive checks, PPCs). Since we only found an effect in choice data, we performed PPCs only for choices (excluding RTs). To this end, we applied a one-step-ahead PPC [25, 30] that factored in participants' actual action and outcome sequences to generate predictions with the entire posterior MCMC samples. Specifically, we let the winning model generate choices as many times as the number of MCMC samples (i.e., 4,000 times) per trial per participant, and we analyzed the generated data the same way as we did for the observed data. We then assessed whether these analyses could reproduce the behavioral pattern in our behavioral analyses (Figure 4B, 4D in the main text).

Simulations of optimal learning rates.

To better understand and interpret the magnitude of the posterior learning rates, we performed simulations with grid approximation to obtain "optimal learning rates", and then compared the estimated posterior learning rates in relation to these optimal parameters (Figure 4A, 4C in the main text). Because there were two learning rates (α^{posPE} and α^{negPE}), to reduce complexity, we fixed the inverse temperature parameter to be the corresponding group-level posterior mean in each condition. For each simulation, we took a small grid per parameter (0:0.01:1) and computed the choice accuracy across 16 trials (identical to the main experiment) for each combination of the parameters. Each simulation was repeated 1000 times to obtain stable results. We then considered the parameters that gave the highest choice accuracy as the optimal learning rates.

Analysis of the RLDDM parameters and their association with prosocial behavior.

Next, we examined whether the behavioral pattern found in the analysis of the correct choice would be associated with differences in the individual model parameters.

As a first step, we tested the parameters of our validated winning model for the 3-way interaction effect of drug treatment, visibility, and type of recipient. There was no significant 3-way interaction in

the learning rate for positive PE (*B* = 1.03, *CI* = [1.00, 1.06], *p* = .063). The analysis of the learning rate for negative PE revealed a three-way interaction of drug treatment, visibility, and type of recipient (*B* = 0.94, *CI* = [0.90, 0.98], *p* = .003) so that the participants in the placebo group had a relatively lower negative learning rate for prosocial choices when being watched than in privacy (recipient x visibility interaction in the placebo group: *B* = 0.68, *CI* = [0.40, 0.96], *p* = .023). Conversely, in the testosterone group, observation (vs privacy) relatively increased the negative learning rate for prosocial choices (recipient x visibility interaction in testosterone group: *B* = 1.21, *CI* = [1.00, 1.40], *p* = .048; Figure 2A). Moreover, the analysis of the choice consistency (inverse temperature parameter tau, described also in the main text) likewise showed a three-way interaction (*B* = 0.98, *CI* = [0.97, 0.98], *p* < .001). Placebo group participants had relatively higher consistency in choices made for the other (vs. self) when being observed than in privacy (recipient x visibility interaction in the placebo group: *B* = 1.09, *CI* = [1.05, 1.14], *p* < .001). On the contrary, in the testosterone group, observation, compared to privacy, decreased the consistency of choices made for the other (vs. self) (recipient x visibility interaction in testosterone group: *B* = 0.90, *CI* = [0.84, 0.98], *p* < .001). When participants were observed, testosterone, compared to placebo, diminished the relative consistency of prosocial choices (recipient x treatment interaction in observed condition: *OR* = 0.91, *CI* = [0.84, 0.99], p =.025). In the private condition, there was no evidence for such an effect (recipient x treatment interaction in private condition: *OR* = 0.99, *CI* = [0.97, 1.02], p = .605).
The analysis of the DDM threshold parameter revealed a three-way interaction as well (*B*= 1.01, *CI* = [1.00, 1.02], *p* < .001; Figure 2C). Placebo group participants had a relatively higher threshold for choices made for another (vs. self) when being observed than in privacy (recipient x visibility interaction in the placebo group: *B* = 1.03, *CI* = [1.01, 1.05], *p* < .001). Conversely, in the testosterone group, observation, compared to privacy, decreased the amount of information required for choices made for another (vs. self) (recipient x visibility interaction in testosterone group: *B* = 0.95, *CI* = [0.93, 0.97], *p* < .001). When participants were observed, testosterone, compared to placebo, decreased the relative threshold of prosocial choices (recipient x treatment interaction in observed condition: *B* = 0.95, *CI* = [0.93, 0.97], p < . 001). The analysis of the DDM drift-scaling parameter revealed a three-way interaction (*B* = 1.01, *CI* = [1.00, 1.02], p < .001). Participants in both placebo (recipient x visibility interaction in placebo group *B* = 0.93, *CI* = [0.91, 0.95], *p* < .001) and testosterone group (recipient x visibility interaction in placebo group *B* = 0.73, *CI* = [0.51, 0.95], *p* < .001) showed relatively lower drift scaling for choices made for another (vs. self) when being observed than in privacy. When participants were observed, testosterone, compared to placebo, decreased the relative drift scaling of prosocial choices (recipient x treatment interaction in the observed group: *B* = 0.91, *CI* = [0.90, 0.92], p < .001).

As a second step, we examined whether the RLDDM parameters that were impacted by testosterone administration predict behavioral prosociality, measured by the difference between correct choices made for other and self across the whole sample. Out of the five parameters, choice consistency (*B* =

3.82, *CI* = [2.64, 5.01], $p < .001$), and DDM threshold ($B = 10.67$, *CI* = [1.57, 19.76], $p = .022$) predicted prosociality, however, only choice consistency survived the Bonferroni correction for multiple comparisons ($p <.01$). Altogether, as reported in the main text, these results suggest that testosterone's impact on strategic prosocial behavior (i.e., audience effect) is strongly linked to testosterone's effect on choice consistency (inverse temperature parameter tau).

Analysis of the drift-scaling parameter and response times.

As specified in equation (7), on each trial *t*, the drift rate v_t was defined with a drift-scaling parameter, v_{scaling} that scales the value difference between the correct and incorrect symbol. Drift-scaling parameter affects the curvature of the function: smaller values lead to a more linear mapping between the value difference and the drift rate, and therefore less sensitivity to value differences.

Drift scaling is conceptually linked to the speed of integration and response times [22], we, therefore, tested whether drift-scaling parameter predicted response times and found a significant association ($B = 0.97$, *CI* = [0.95, 0.98], $p < .001$). However, contrary to correct choices, response times did not differ across experimental groups (drug treatment: $B = 1.01$, *CI* = [0.97, 1.05], p = .737; visibility: $B = 0.98$, *CI* = [0.94, 1.02], $p < .265$; recipient: $B = 0.99$, *CI* = [0.99, 1.00], $p < .106$; drug treatment x visibility x recipient: $B = 1.00$, *CI* = [0.99, 1.01], $p < .806$).

Supplementary information on the analysis of genetic data

Previous research suggested that testosterone may influence behavior through dopaminergic pathway [31]. In humans, testosterone administration enhanced activation of the ventral striatum to monetary rewards [32] and the enhancing effects of exogenous testosterone on competitive status-seeking were more pronounced among individuals with a 9/10R compared to 10/10R genotype of the dopamine transporter (DAT) [33]. The expression of DAT, which regulates striatal dopamine, is linked to a 40 base-pair variable number tandem repeat polymorphism of the DAT1 gene [34]. Homozygous 10/10-repeat carriers of this polymorphism have higher DAT expression (i.e., lower striatal dopamine) than heterozygous, 9-repeat variant, individuals [34].

Testosterone's effects on status-seeking behavior have likewise been shown to be enhanced among individuals with fewer CAG repeats in exon 1 of the androgen-receptor gene [33,36]. In-vitro experimental work suggests that increasing the number of CAG repeats within the androgen receptor (AR) gene reduces the receptor's transcriptional potential [37]. In other words, the efficiency of the androgen receptors is negatively related to the CAG repeat [38].

We, therefore, tested whether testosterone's effects on strategic prosociality depended on individual differences in striatal dopamine, assessed by DAT1 polymorphism, and the efficiency of ARs, assessed by the CAG repeat polymorphism.

Genotyping of AR CAG repeat and DAT1 polymorphisms.

DNA was extracted from buccal swabs and isolated using a resin-based method with Chelex®100 (Sigma Aldrich, USA). For amplification of the CAG repeat polymorphism in exon 1 of the AR gene primers forward - 5' GCGCGAAGTGATCCAGAAC 3' tagged with 6–carboxyfluorescein and reverse - 5' CTCATCCAGGACCAGGTAGC 3', and for amplification of the DAT1-3'UTR VNTR polymorphism primers forward - 5' GTCCTTGTGGTGTAGGGAAC 3' tagged with 6–carboxyfluorescein and reverse - 5' CTGGAGGTCACGGCTCAAG 3' were used in PCR with 20 µL reaction using 250 nmol/L final primer molarity. As PCR masterrmix 5x Hot FIREPol Blend Mastermix with 7.5 mM MgCl2 (Solis Biodyne, Tartu, Estonia) was used in all amplifications. The following PCR program was used: initial denaturation step at 95°C for 15 min, followed by 30 cycles each consisting of denaturation at 95°C for 30 s, annealing at 60°C for 30 s and polymerization at 72°C for 1 min. The number of repeats of AR CAG STR and DAT1-3'UTR VNTR was analyzed by fragment analysis using Sanger sequencing on ABI 3500 Genetic Analyzer (Applied Biosystems, USA).

Interaction of DAT1 polymorphism with testosterone effects on the correct choice and RLDDM parameters.

There were no significant differences in the distribution of the genotype among our experimental groups (χ^2 (6, *N* = 190) = 5.76 *p* = .451). The 9/10R and the 10/10R genotypes accounted for most of the observed DAT1 genotypes in our sample (36% (N=68) and 56% (N=105), respectively), and we thus used these two genotypes in the analyses by adding DAT1 polymorphisms as a predictor in interaction with the other factors (recipient, drug treatment, visibility) to the GzLMM of correct choice and the GzLMMs of the RLDDM parameters (see *Materials and Methods* in the main manuscript text). The analysis revealed no significant interaction of DAT1 polymorphism with testosterone effect on correct choice (recipient x drug treatment x visibility x DAT1: *OR* = 1.08, *CI* = [0.95, 1.23], *p* = .258), α^{posPE} (recipient x drug treatment x visibility x DAT1: *B* = 0.98, *CI* = [0.92, 1.05], *p* = .578), α^{negPE} (recipient x drug treatment x visibility x DAT1: *B* = 1.07, *CI* = [0.98, 1.17], *p* = .141), choice consistency (recipient x drug treatment x visibility x DAT1: *B* = 1.01, *CI* = [1.00, 1.02], *p* = .091) or decision threshold (recipient x drug treatment x visibility x DAT1: *B* = 1.00, *CI* = [1.00, 1.00], *p* = .986).

Interaction of AR CAG repeat polymorphism with testosterone effects on the correct choice and RLDDM parameters.

Mean-centered CAG repeat lengths of the AR gene in exon 1 were included as a predictor in interaction with the other factors (recipient, drug treatment, visibility) to the GzLMM of correct choice and the GzLMMs of the RLDDM parameters (see *Materials and Methods* in the main text). The analysis revealed no significant interaction of CAG repeat polymorphism with testosterone effect

on correct choice (recipient x drug treatment x visibility x CAG: *OR* = 1.00, *CI* = [0.99, 1.02], *p* = .599), α^{posPE} (recipient x drug treatment x visibility x CAG: *B* = 1.00, *CI* = [1.00, 1.01], *p* = .363), αneg^{PE} (recipient x drug treatment x visibility x CAG: *B* = 1.01, *CI* = [0.99, 1.01], *p* = .243), choice consistency (recipient x drug treatment x visibility x CAG: *B* = 1.00, *CI* = [0.99, 1.01], *p* = .576) or decision threshold (recipient x drug treatment x visibility x CAG: *B* = 1.00, *CI* = [1.00, 1.00], *p* = .103).

Interaction of trait dominance with testosterone effects on RLDDM parameters.

Mean-centered dominance scores [39] were included as a predictor in interaction with the other factors (recipient, drug treatment, visibility) to the GzLMM of correct choice (reported in the main text) and the GzLMMs of the RLDDM parameters. Contrary to the former, the latter analysis revealed no significant interaction of dominance scores with testosterone effect RLDDM parameters: learning rate for positive PE (recipient x drug treatment x visibility x dominance: *B* = 1.01, *CI* = [0.97, 1.04], *p* = .743), learning rate for negative PE (recipient x drug treatment x visibility x dominance: *B* = 1.02, *CI* = [0.98, 1.07], *p* = .331), choice consistency (recipient x drug treatment x visibility x dominance: *B* = 1.00, *CI* = [0.99, 1.02], *p* = .644), or decision threshold (recipient x drug treatment x visibility x dominance: *B* = 1.00, *CI* = [1.00, 1.00], *p* = .578).

Supplementary information on the questionnaire data

Post-task questionnaire.

To estimate the subjective perception of being watched, a post-task questionnaire was administered immediately after the end of the reinforcement-learning paradigm. The participants were asked the question: "Did you feel that you were being watched while performing the task?" The answers were classified into three categories: 1 (Not at all), 2 (Moderately), and 3 (Strongly). First, we investigated whether the subjective feelings of being watched were related to the cortisol reactivity to visibility manipulation (see *Manipulation check* in the manuscript's main text). We next examined whether the subjective feelings of being watched interact with the testosterone administration effect on prosocial choice and RLDDM parameters. As the factors *visibility* and *subjective feeling of being watched* are not orthogonal, we did not add the subjective feelings of being watched as a fourth factor to the analysis. Instead, we replaced the factor *visibility* with the factors *subjective feeling of being watched* and compared whether such a model explains more variance than the original one. We did not find a significant interaction of the factors administration x recipient x subjective feeling of being watched (*OR* = 0.89, *CI* = [0.79, 1.01], *p* = .133). This model did not explain more variance (*Conditional R2*=.102) than the original administration x recipient x visibility model (*Conditional R2*=.103).

Portrait Values Questionnaire.

We conducted exploratory analyses to examine what motivational constructs may have interacted with testosterone's effects. To this end, we analyzed data from the Portrait Values Questionnaire [40, 41] which had been administered as part of the study's extensive questionnaire battery. Note that these are exploratory analyses and that this specific questionnaire was originally intended to be used for analyses related to other tasks that were part of the overall study. They should therefore be considered with caution, and we have clearly labeled them as exploratory and post-hoc. In brief, the Portrait Values Questionnaire is designed to capture the structure of human values explaining the motivational bases of our attitudes and behavior. The questionnaire allowed us to distinguish four principal value orientations: self-transcendence, self-enhancement, conservation, and openness. The four value subscales were separately added to our analysis of recipient x administration x visibility interaction as mean-centered interaction terms. This revealed a significant interaction of *self-enhancement value orientation* with testosterone's effect on the number of correct choices (recipient x visibility x administration x self-enhancement*:* $OR = 0.65$, $CI = [0.48, 0.88]$, $p = .006$) and interaction of *conservation value orientation* and testosterone's effects on choice consistency (recipient x visibility x administration x conservation: $B = 0.60$, $CI = [0.17, 1.02]$, $p = .006$). The follow-up analyses of these significant interactions are reported in the main manuscript text.

Self-enhancement value did not significantly interact with testosterone effects on choice consistency (recipient x drug treatment x visibility x self-enhancement: $B = -0.01$, $CI = [-0.06, 0.04]$, $p = .633$). Conservation did not significantly interact with testosterone effects on correct choice (recipient x drug treatment x visibility x conservation: $OR = 0.98$, $CI = [0.94, 1.02]$, $p = .287$). Self-transcendence did not significantly interact with testosterone effects on correct choice (recipient x drug treatment x visibility x self-transcendence: $OR = 0.97$, $CI = [0.91, 1.03]$, $p = .319$) or choice consistency (recipient x drug treatment x visibility x self-transcendence: $B = 0.33$, $CI = [-0.34, 1.00]$, $p = .337$). Lastly, openness value did not significantly interact with testosterone effects on correct choice (recipient x drug treatment x visibility x openness: $OR = 1.02$, $CI = [0.98, 1.07]$, $p = .328$) or choice consistency (recipient x drug treatment x visibility x openness: $B = -0.01$, $CI = [-0.07, 0.04]$, $p = .646$).

Observers' salience survey.

To estimate the salience of the observers introduced as NGO representatives in our paradigm, we conducted an additional online survey. Using Amazon Mechanical Turk (MTruk) we interviewed N = 73 volunteers (sample size determined by G*Power [42] to achieve 95 % power to detect a small effect size $f = .20$) aged $M = 34.55$ ($SD = 9.12$) years. Participants were asked to perform the task alone and rate how much they agree with the statements regarding different types of observers. Together they rated 7 statements with the following wording, and each statement concerned a different type of observer:

“As I perform this task on my computer/mobile device alone, I anticipate I would feel differently if ……… would watch me perform this task.”

The observer’s types were: an unknown other, a friend, a parent, a shop assistant, an NGO representative, a colleague from work, and a technical support worker. A repeated-measures ANOVA comparing the ratings of the respective observers showed that there was a difference across the levels of the within-subject factor $F(6, 396) = 2.69$, $p = .014$, $f = .204$. A follow-up analysis using treatment contrasts and the NGO as a reference level showed a significant difference between the levels of NGO representative and parent ($B = -0.57$, $p < 0.001$), meaning that participants would feel different to a greater extent if they were watched by an NGO representative than a parent. Crucially, no other pairwise comparisons were significant, all *p*s >.169).”

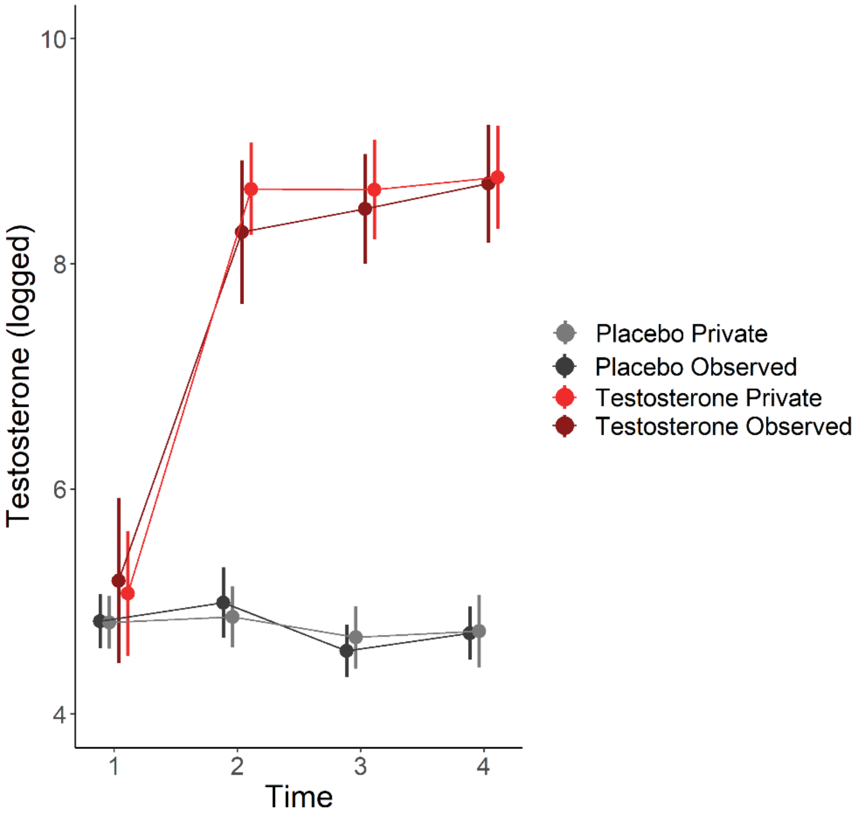

Figure S1. Testosterone levels during the experimental session. Error bars = Mean ± 95%CI.

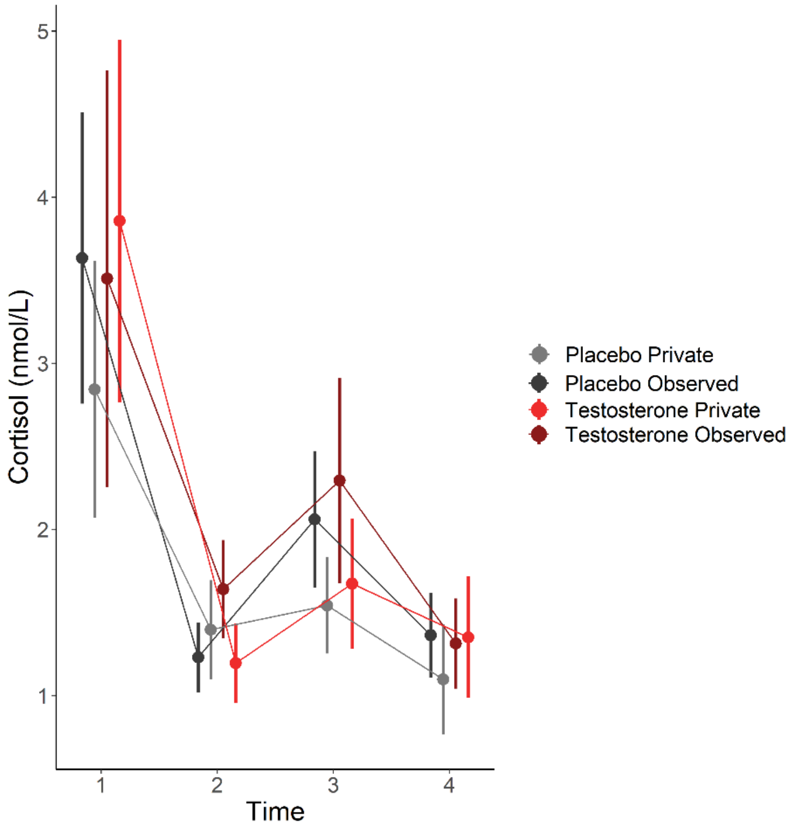

Figure S2. Cortisol levels during the experimental session. Error bars = Mean ± 95%CI

Table S1. Summary statistics across experimental groups: Mean (95%CI), ANOVA.

**Drug treatment**	**Placebo**		**Testosterone**			
**Visibility**	**Private**	**Observed**	**Private**	**Observed**	**F**	**p**
N	47	45	53	45		
Age	25.4 (24.5, 26.3)	24.5 (23.3, 25.7)	24.0 (23.1, 24.9)	25.1 (24.0, 26.2)	1.140	.239
Baseline testosterone [pg/mL]	143 (76.7, 209)	142 (74.9, 209)	225 (153.5, 296)	268 (180.9, 354)		
log	4.75 (4.52, 4.98)	4.76 (4.53, 5.00)	4.94 (4.69, 5.19)	4.99 (4.69, 5.30)	0.024	.877
Baseline cortisol [nmol/L]	2.84 (1.81, 3.87)	3.67 (2.64, 4.71)	3.89 (2.93, 4.86)	3.56 (2.52, 4.61)		
log	0.77 (0.52, 1.02)	0.98 (0.73, 1.24)	0.90 (0.65, 1.12)	0.86 (0.60, 1.11)	0.930	.336
Baseline estradiol [pg/mL]	3.68 (3.12, 4.23)	3.76 (3.20, 4.31)	3.71 (3.19, 4.22)	3.75 (3.19, 4.31)		
log	1.23 (1.09, 1.36)	1.20 (1.06, 1.33)	1.19 (1.07, 1.32)	1.23 (1.09, 1.36)	0.194	.660
CAG-repeat polymorphism	19.7 (18.7, 20.7)	19.8 (18.8, 20.8)	19.3 (18.4, 20.3)	19.3 (18.4, 20.5)	0.001	.999
Dominance	3.90 (3.63, 4.17)	4.02 (3.74, 4.30)	4.04 (3.78, 4.30)	3.82 (3.55, 4.10)	1.526	.218

Table S2. Descriptive statistics of cortisol levels during the experiment.

Drug treatment	Placebo						Testosterone					
Visibility	Private			Observed			Private			Observed		
	Mean (CI)	Min	Max	Mean (CI)	Min	Max	Mean (CI)	Min	Max	Mean (CI)	Min	Max
Time 1	2.84 (1.81, 3.87)	0.64	15.34	3.67 (2.64, 4.71)	0.37	12.87	3.89 (2.93, 4.86)	0.23	18.59	3.56 (2.52, 4.61)	0.44	23.80
Time 2	1.39 (1.13, 1.66)	0.34	5.14	1.22 (0.96, 1.49)	0.22	3.55	1.19 (0.95, 1.45)	0.17	4.40	1.63 (1.33, 1.93)	0.29	3.98
Time 3	1.54 (1.11, 1.98)	0.38	3.94	2.05 (1.62, 2.50)	0.51	6.49	1.67 (1.27, 2.08)	0.28	9.12	2.29 (1.85, 2.74)	0.30	11.62
Time 4	1.09 (0.78, 1.42)	0.21	5.73	1.36 (1.05, 1.67)	0.47	4.86	1.31 (1.06, 1.64)	0.15	6.57	1.35 (0.99, 1.63)	0.15	3.77

Table S3. Model space and model evidence.

RW, Rescorla-Wagner model; DLR dual learning rates model; DDM, drift diffusion model; RLDDM, reinforcement learning drift diffusion model; RLDDM-nonlinear, RLDDM with a non-linear transformation function; LOOIC leave-one-out information criterion (lower LOOIC value indicates better out-of-sample predictive accuracy); weight, model weight calculated with Bayesian model averaging using Bayesian bootstrap (higher model weight value indicates a higher probability of the candidate model to have generated the observed data). The winning model is highlighted in bold. Because LOOIC provides relative quantifications of model performance, and the data to be modeled varied across conditions, the absolute magnitude of LOOIC should not be interpreted.

**Task condition** / **Model space**		**Placebo Observed**		**Placebo Private**		**Testosterone observed**		**Testosterone Private**	
		LOOIC	Weight	LOOIC	Weight	LOOIC	Weight	LOOIC	Weight
RW	DDM	9688	0	10939	0	10202	0	12716	0
	RLDDM	8365	0	9747	0	9135	0	11450	0
	RLDDM-nonlinear	8235	0	9611	0	8984	0	11184	0
DLR	DDM	9538	0	10709	0	10018	0	12487	0
	RLDDM	7877	0.004	9081	0.3	8643	0.002	10688	0
	**RLDDM-nonlinear**	**7832**	**0.996**	**9040**	**0.97**	**8352**	**0.998**	**10516**	**1**

Table S4. Summary of parameters in the winning model.

Note that all parameters were further separated for "self" versus "other", hence for each between-subject condition, the winning model contained 14 parameters. The initial bias *z* in DDM was fixed at 0.5. min(RT), lowest response time from observed data.

Component	Parameter	Meaning	Interpretation
RL	$0 < \alpha^{posPE} < 1$	learning rate for updating from positive reward prediction error	the learning rate weighs the effect of the reward prediction error in the value update; a higher (lower) learning rate means a faster (slower) value update from the most recent outcome
	$0 < \alpha^{negPE} < 1$	learning rate for updating from negative reward prediction error	
	$\beta > 0$	inverse temperature in softmax action selection function	choice consistency parameter, captures how much choices rely on the value updates
DDM	$v_{max} > 0$	maximum value of the drift rate in the non-linear function	defines the upper/lower boundaries in the sigmoid non-linear function
	$v_{scaling} > 0$	drift scaling that maps value difference into the drift rate	scales the effect of value difference between the choice options on the drift rate
	$a > 0$	decision threshold, i.e., the distance between choice alternatives	"the endpoint" of the evidence accumulation process, captures the amount of information necessary to make a decision
	$0 < T < \min(RT)$	non-decision time	considered to capture sensory delay and/or movement initiation

SM References

1. R Core Team. R: A Language and Environment for Statistical Computing. Vienna, Austria: R Foundation for Statistical Computing; 2020.

2. Bates D, Mächler M, Bolker B, Walker S. Fitting Linear Mixed-Effects Models Using lme4. Journal of Statistical Software. 2015;67:1–48.

3. Fox J, Weisberg S. An R Companion to Applied Regression. Third. Thousand Oaks CA: Sage; 2019.

4. Lüdecke D. sjPlot: Data Visualization for Statistics in Social Science. 2020.

5. Phillips N. yarrr: A Companion to the e-Book 'YaRrr!: The Pirate's Guide to R'. 2017.

6. Wickham H. ggplot2: Elegant Graphics for Data Analysis. Springer-Verlag New York; 2016.

7. Carpenter B, Gelman A, Hoffman MD, Lee D, Goodrich B, Betancourt M, et al. Stan: A Probabilistic Programming Language. Journal of Statistical Software. 2017;76:1–32.

8. Ahn W-Y, Haines N, Zhang L. Revealing Neurocomputational Mechanisms of Reinforcement Learning and Decision-Making With the hBayesDM Package. Comput Psychiatr. 2017;1:24–57.

9. Vehtari A, Gelman A, Gabry J. Practical Bayesian model evaluation using leave-one-out cross-validation and WAIC. ArXiv:150704544 [Stat]. 2016. 12 September 2016. https://doi.org/10.1007/s11222-016-9696-4.

10. Knight EL, McShane BB, Kutlikova HH, Morales PJ, Christian CB, Harbaugh WT, et al. Weak and Variable Effects of Exogenous Testosterone on Cognitive Reflection Test Performance in Three Experiments: Commentary on Nave, Nadler, Zava, and Camerer (2017). Psychol Sci. 2020;31:890–897.

11. Nave G, Nadler A, Zava D, Camerer C. Single-Dose Testosterone Administration Impairs Cognitive Reflection in Men. Psychol Sci. 2017;28:1398–1407.

12. Wang C, Plymate S, Nieschlag E, Paulsen CA. Salivary testosterone in men: further evidence of a direct correlation with free serum testosterone. J Clin Endocrinol Metab. 1981;53:1021–1024.

13. R. A. Rescorla, A. R. Wagner, "A theory of Pavlovian conditioning: Variations in the effectiveness of reinforcement and nonreinforcement" in *Classical Conditioning II: Current Research and Theory*, (Appleton-Century-Crofts, 1972), pp. 64–99.

14. Gershman SJ. Do learning rates adapt to the distribution of rewards? Psychon Bull Rev. 2015;22:1320–1327.

15. Pedersen ML, Frank MJ, Biele G. The drift diffusion model as the choice rule in reinforcement learning. Psychon Bull Rev. 2017;24:1234–1251.

16. Cox SML, Frank MJ, Larcher K, Fellows LK, Clark CA, Leyton M, et al. Striatal D1 and D2 signaling differentially predict learning from positive and negative outcomes. Neuroimage. 2015;109:95–101.

17. Frank MJ, Moustafa AA, Haughey HM, Curran T, Hutchison KE. Genetic triple dissociation reveals multiple roles for dopamine in reinforcement learning. Proc Natl Acad Sci U S A. 2007;104:16311–16316.

18. den Ouden HEM, Daw ND, Fernandez G, Elshout JA, Rijpkema M, Hoogman M, et al. Dissociable effects of dopamine and serotonin on reversal learning. Neuron. 2013;80:1090–1100.

19. Crawley D, Zhang L, Jones EJH, Ahmad J, Oakley B, Cáceres ASJ, et al. Modeling flexible behavior in childhood to adulthood shows age-dependent learning mechanisms and less optimal learning in autism in each age group. PLOS Biology. 2020;18:e3000908.

20. Lengersdorff LL, Wagner IC, Lockwood PL, Lamm C. When Implicit Prosociality Trumps Selfishness: The Neural Valuation System Underpins More Optimal Choices When Learning to Avoid Harm to Others Than to Oneself. J Neurosci. 2020;40:7286–7299.

21. Lefebvre G, Lebreton M, Meyniel F, Bourgeois-Gironde S, Palminteri S. Behavioural and neural characterization of optimistic reinforcement learning. Nat Hum Behav. 2017;1:1–9.

22. Ratcliff R, McKoon G. The Diffusion Decision Model: Theory and Data for Two-Choice Decision Tasks. Neural Comput. 2008;20:873–922.

23. Navarro DJ, Fuss IG. Fast and accurate calculations for first-passage times in Wiener diffusion models. Journal of Mathematical Psychology. 2009;53:222–230.

24. Fontanesi L, Gluth S, Spektor MS, Rieskamp J. A reinforcement learning diffusion decision model for value-based decisions. Psychon Bull Rev. 2019;26:1099–1121.

25. Zhang L, Gläscher J. A brain network supporting social influences in human decision-making. Science Advances. 2020;6:eabb4159.

26. Gelman A, Carlin JB, Stern HS, Dunson DB, Vehtari A, Rubin DB. Bayesian Data Analysis. 3rd ed. New York: Chapman and Hall/CRC; 2015.

27. Gelman A, Rubin DB. Inference from Iterative Simulation Using Multiple Sequences. Statistical Science. 1992;7:457–472.

28. Vehtari A, Gelman A, Gabry J. Practical Bayesian model evaluation using leave-one-out cross-validation and WAIC. ArXiv:150704544 [Stat]. 2016. 12 September 2016. https://doi.org/10.1007/s11222-016-9696-4.

29. Yao Y, Vehtari A, Simpson D, Gelman A. Using Stacking to Average Bayesian Predictive Distributions (with Discussion). Bayesian Analysis. 2018;13:917–1007.

30. Zhang L, Lengersdorff L, Mikus N, Gläscher J, Lamm C. Using reinforcement learning models in social neuroscience: frameworks, pitfalls and suggestions of best practices. Social Cognitive and Affective Neuroscience. 2020;15:695–707.

31. Purves-Tyson TD, Owens SJ, Double KL, Desai R, Handelsman DJ, Weickert CS. Testosterone Induces Molecular Changes in Dopamine Signaling Pathway Molecules in the Adolescent Male Rat Nigrostriatal Pathway. PLoS One. 2014;9:e91151.

32. Hermans EJ, Bos PA, Ossewaarde L, Ramsey NF, Fernández G, van Honk J. Effects of exogenous testosterone on the ventral striatal BOLD response during reward anticipation in healthy women. Neuroimage. 2010;52:277–283.
33. Losecaat Vermeer AB, Krol I, Gausterer C, Wagner B, Eisenegger C, Lamm C. Exogenous testosterone increases status-seeking motivation in men with unstable low social status. Psychoneuroendocrinology. 2020;113:104552.

34. Vandenbergh DJ, Persico AM, Hawkins AL, Griffin CA, Li X, Jabs EW, et al. Human dopamine transporter gene (DAT1) maps to chromosome 5p15.3 and displays a VNTR. Genomics. 1992;14:1104–1106.

35. Heinz A, Goldman D, Jones DW, Palmour R, Hommer D, Gorey JG, et al. Genotype Influences In Vivo Dopamine Transporter Availability in Human Striatum. Neuropsychopharmacol. 2000;22:133–139.

36. Geniole SN, Procyshyn TL, Marley N, Ortiz TL, Bird BM, Marcellus AL, et al. Using a Psychopharmacogenetic Approach To Identify the Pathways Through Which—and the People for Whom—Testosterone Promotes Aggression. Psychol Sci. 2019;30:481–494.

37. Chamberlain NL, Driver ED, Miesfeld RL. The length and location of CAG trinucleotide repeats in the androgen receptor N-terminal domain affect transactivation function. Nucleic Acids Res. 1994;22:3181–3186.

38. Zitzmann M, Nieschlag E. The CAG repeat polymorphism within the androgen receptor gene and maleness. Int J Androl. 2003;26:76–83.

39. Cheng JT, Tracy JL, Henrich J. Pride, personality, and the evolutionary foundations of human social status. Evolution and Human Behavior. 2010;31:334–347.

40. Schwartz SH. Chapter 7 A Proposal for Measuring Value Orientations across Nations 2007.

41. Schwartz, SH, Breyer, B, & Danner, D. Human Values Scale (ESS). Zusammenstellung sozialwissenschaftlicher Items und Skalen (ZIS) 2015.

42. Faul F, Erdfelder E, Lang A-G, Buchner A. G*Power 3: a flexible statistical power analysis program for the social, behavioral, and biomedical sciences. Behav Res Methods. 2007;39:175–191.
